# Taxonomy and phylogeny of the *Leptographium olivaceum* complex (Ophiostomatales, Ascomycota), including descriptions of six new species from China and Europe

**DOI:** 10.3897/mycokeys.60.39069

**Published:** 2019-11-29

**Authors:** Mingliang Yin, Michael J. Wingfield, Xudong Zhou, Riikka Linnakoski, Z. Wilhelm de Beer

**Affiliations:** 1 Guangdong Key Laboratory for Innovative Development and Utilization of Forest Plant Germplasm, College of Forestry and Landscape Architecture, South China Agricultural University, Guangzhou, 510000, China; 2 Department of Microbiology and Plant Pathology, Forestry and Agricultural Biotechnology Institute (FABI), University of Pretoria, Pretoria 0002, Gauteng Province, South Africa; 3 FuturaGene Biotechnology (Shanghai) Co. Ltd, Shanghai, 200233, China; 4 Natural Resources Institute Finland (Luke), 00790 Helsinki, Finland

**Keywords:** bark beetle, *
Leptographium
*, integrative taxonomy, new species, *
Ophiostomatales
*, phylogeny

## Abstract

The *Leptographium olivacea* complex encompasses species in the broadly defined genus *Leptographium* (Ophiostomatales, Ascomycota) that are generally characterized by synnematous conidiophores. Most species of the complex are associates of conifer-infesting bark beetles in Europe and North America. The aims of this study were to reconsider the delineation of known species, and to confirm the identity of several additional isolates resembling *L. olivacea* that have emerged from recent surveys in China, Finland, Poland, Russia, and Spain. Phylogenetic analyses of sequence data for five loci (ACT, TUB, CAL, ITS2-LSU, and TEF-1α) distinguished 14 species within the complex. These included eight known species (*L. cucullatum*, *L. davidsonii*, *L. erubescens*, *L. francke-grosmanniae*, *L. olivaceum*, *L. olivaceapini*, *L. sagmatosporum*, and *L. vescum*) and six new species (herein described as *L. breviuscapum*, *L. conplurium*, *L. pseudoalbum*, *L. rhizoidum*, *L. sylvestris*, and *L. xiningense*). New combinations are provided for *L. cucullatum*, *L. davidsonii*, *L. erubescens*, *L. olivaceum*, *L. olivaceapini*, *L. sagmatosporum* and *L. vescum*. New Typifications: Lectotypes are designated for *L. olivaceum*, *L. erubescens* and *L. sagmatosporum*. Epitypes were designated for *L. olivaceapini* and *L. sagmatosporum*. In addition to phylogenetic separation, the synnematous asexual states and ascomata with almost cylindrical necks and prominent ostiolar hyphae, distinguish the *L. olivaceum* complex from others in *Leptographium*.

## Introduction

Species of *Leptographium* are commonly associated with bark beetles and weevils, and are responsible for causing sapstain on a wide range of primarily coniferous trees (Jacobs and Wingfield 2001). The genus also includes some important tree pathogens such as species in the *Leptographium wageneri* complex that cause black stain root disease (Goheen and Hansen 1978). In their monograph of *Leptographium*, Jacobs and Wingfield (2001) treated the asexual states of 46 species in the genus, all characterized by mononematous conidiophores branched at their apices. Conidia aggregate in slimy droplets at the apices of these structures, which make these species well-adapted for arthropod dispersal.

Following the “one fungus one name” principles adopted in the Melbourne Code (Hawksworth 2011), De Beer and Wingfield (2013) re-evaluated the taxonomy of *Leptographium*, considering available DNA sequence data for all species. Ninety-four species were included and ten species complexes were defined within a broadly defined concept for *Leptographiumsensu lato*, based on phylogenies resulting from ribosomal internal transcribed spacer (**ITS**) and partial LSU sequences.

One of the species complexes recognized in *Leptographium* s.l. by De Beer and Wingfield (2013) was the *L. olivaceum* complex. Earlier, Zipfel et al. (2006) had shown that *L. olivaceum* produces synnematous asexual states, which is unlike mononematous conidiophores traditionally defining *Leptographium*. In extended phylogenies, Massoumi Alamouti et al. (2007), Six et al. (2011), and Linnakoski et al. (2012) showed that additional species with synnematous asexual states grouped in a monophyletic lineage with *L. olivaceum.*Six et al. (2011) referred to this lineage as the *L. olivaceum* species complex for the first time and they included *L. olivaceum* (Mathiesen-Käärik, 1951), *L. sagmatosporum* (Wright & Cain, 1961), *L. olivaceapini* (Davidson, 1971), and *L. cucullatum* (Solheim, 1986) in their phylogeny. Subsequently, *L. davidsonii* (Olchowecki & Reid, 1974) and *L. vescum* (Davidson, 1958) were shown to also belong to this complex (Linnakoski et al. 2012, De Beer and Wingfield 2013).

The six species currently residing in the *L. olivaceum* complex have morphologically similar sexual and asexual states. They produce globose ascomata with long, nearly cylindrical necks, terminating in prominent ostiolar hyphae on which sticky droplets are formed that contain orange-section shaped ascospores with cucullate gelatinous sheaths (Mathiesen-Käärik 1951, Davidson 1958, Wright and Cain1961, Davidson 1971, Olchowecki and Reid 1974, Solheim 1986). This study includes isolates representing all species in the *L. olivaceum* complex as well as morphologically similar isolates from recent surveys of fungi in China, Europe, and Russia. The aims of the study were to reconsider and redefine the species boundaries in the *L. olivaceum* complex based on phylogenetic analyses of multilocus regions, to provide neotypes for species where type specimens have been lost or are inadequate, and to describe new species in this complex.

## Methods

### Isolates

All isolates included in this study are listed in Table 1. Reference isolates were obtained from the culture collection (**CMW**) of the Forestry and Agricultural Biotechnology Institute (**FABI**), University of Pretoria, South Africa. Ex-type isolates of newly described species were deposited in the Westerdijk Fungal Biodiversity Institute (**CBS**), Utrecht, in the Netherlands. Type specimens of new species were deposited in the National Collection of Fungi (**PREM**), Pretoria, South Africa. Taxonomic novelties and new typification events for known taxa were registered in MycoBank (Robert et al. 2013).

**Table 1.** Isolates used in the present study.

**Species^1^**	**Isolate no.^2^**		**Country**	**Host**	**Insect**	**GenBank accession no. ^3^**				
	**CMW no.**	**CBS no.**				**ITS2-LSU**	**ACT**	**TUB**	**CAL**	**TEF-1α**
***Leptographium breviscapum***	38888 ^H^	136507	China	*Picea crassifolia*	*Polygraphus poligraphus*	**MN516697**	**MN517641**	**MN517672**	**MN517707**	**MN517742**
	38889 ^P^	136508	China	*Picea crassifolia*	*Polygraphus poligraphus*	**MN516698**	**MN517642**	**MN517673**	**MN517708**	**MN517743**
	38890		China	*Picea crassifolia*	*Polygraphus poligraphus*	**MN516699**	**MN517643**	**MN517674**	**MN517709**	**MN517744**
***L. conplurium***	23289 ^P^	128834	Finland	*Picea abies*	*Dryocoetus autographus*	**MN516701**	**MN517644**	JF279994	**MN517710**	**MN517745**
	23295		Finland	*Picea abies*	*Dryocoetus autographus*	**MN516702**	**MN517645**	JF279993	**MN517711**	JF280036
	23315 ^H^	128923	Finland	*Picea abies*	*Dryocoetus autographus*	**MN516700**	**MN517646**	JF279989	**MN517712**	**MN517746**
	23316		Finland	*Picea abies*	*Hylastes brunneus*	**MN516703**	**MN517647**	JF279990	**MN517713**	**MN517747**
*L. cucullatum*	1140=1141 ^H^	218.83	Norway	*Picea abies*	*Ips typographus*	AJ538335	**MN517619**	JF280000	**MN517685**	**MN517724**
	1871		Japan	*Pinus jezoensis*	*Ips typographus*	**MN516704**	**MN517620**	JF280001	**MN517686**	**MN517725**
	5022		Austria	*Picea abies*	*Ips typographus*	**MN516705**	**MN517621**	JF280002	**MN517687**	**MN517726**
	23123	128299	Russia	*Picea abies*	*Ips typographus*	**MN516706**	**MN517622**	JF280003	**MN517688**	JF280042
	23190		Russia	*Pinus sylvestris*	*Ips typographus*	**MN516707**	**MN517623**	JF280005	**MN517689**	JF280043
	27983		Russia	*Picea abies*	*Dryocoetus autographus*	**MN516708**	**MN517624**	**MN517658**	**MN517690**	**MN517727**
	27984		Russia	*Picea abies*	*Dryocoetus autographus*	**MN516709**	**MN517625**	**MN517659**	**MN517691**	**MN517728**
	36623		Russia	*Picea abies*	*Ips typographus*	**MN516710**	**MN517626**	**MN517660**	**MN517692**	**MN517729**
*L. davidsonii*	790 ^H^		Canada	*Pseudotsuga menziesii*	–	**MN516711**	**MN517627**	**MN517661**	**MN517693**	**MN517730**
	3094		Canada	*Picea* sp.	unknown bark beetle	**MN516712**	**MN517628**	**MN517662**	**MN517694**	**MN517731**
	3095		Canada	*Picea* sp.	unknown bark beetle	**MN516713**	**MN517629**	**MN517663**	**MN517695**	**MN517732**
*L. erubescens*	40672 ^H^	278.54	Sweden	*Pinus sylvestris*	–	**MN516714**	**MN517656**	**MN517683**	**MN517722**	**MN517756**
*L. francke-grosmanniae*	445 ^H^	356.77	Germany	*Quercus* sp.	*Hylecoetus dermestoides*	**MN516715**	**MN517618**	**MN517657**	**MN517684**	**MN517723**
*L. olivaceum*	23348	128836	Finland	*Picea abies*	*Ips typographus*	**MN516717**	**MN517630**	**MN517664**	**MN517696**	JF280049
	23350	128837	Finland	*Picea abies*	*Ips typographus*	**MN516718**	**MN517631**	**MN517665**	**MN517697**	JF280050
	28090		Russia	*Pinus sylvestris*	*Ips typographus*	**MN516719**	**MN517632**	**MN517666**	**MN517698**	**MN517733**
	31059 ^H^	138.51	Sweden	*Pinus sylvestris*	–	**MN516716**	**MN517633**	JF279997	**MN517699**	**MN517734**
	31060	152.54	Sweden	–	–	**MN516720**	**MN517634**	JF279998	**MN517700**	**MN517735**
*L. olivaceapini*	63	503.86	USA	–	–	**MN516721**	**MN517635**	**MN517667**	**MN517701**	**MN517736**
	116 ^E^	504.86	USA	–	–	**MN516722**	**MN517636**	**MN517668**	**MN517702**	**MN517737**
***L. pseudoalbum***	40671 ^H^	276.54	Sweden	*Pinus sylvestris*	*Tomicus piniperda*	**MN516723**	**MN517655**	**MN517682**	**MN517721**	**MN517755**
***L. rhizoidum***	22809 ^H^	136512	Spain	*Pinus radiata*	*Hylastes ater*	**MN516724**	**MN517648**	**MN517675**	**MN517714**	**MN517748**
	22810 ^P^	136513	Spain	*Pinus radiata*	*Hylastes attenuatus*	**MN516725**	**MN517649**	**MN517676**	**MN517715**	**MN517749**
	22811		Spain	*Pinus radiata*	*Ips sexdentatus*	**MN516726**	**MN517650**	**MN517677**	**MN517716**	**MN517750**
	22812		Spain	*Pinus radiata*	*Hylurgops palliatus*	**MN516727**	**MN517651**	**MN517678**	**MN517717**	**MN517751**
*L. sagmatosporum*	34135 ^E^	113452	Canada	*Pinus strobus*	–	**MN516728**	**MN517637**	**MN517669**	**MN517703**	**MN517738**
***L. sylvestris***	23300 ^P^	128833	Finland	*Picea abies*	*Ips typographus*	**MN516729**	**MN517639**	JF279996	**MN517705**	**MN517740**
	34140 ^T^	136511	Poland	*Pinus sylvestris*	–	**MN516730**	**MN517640**	**MN517671**	**MN517706**	**MN517741**
*L. vescum*	34186 ^H^	800.73	USA	*Picea engelmannii*	*Ips pilifrons, Dendroctonus engelmanni*	**MN516731**	**MN517638**	**MN517670**	**MN517704**	**MN517739**
***L. xiningense***	38891 ^H^	136509	China	*Picea crassifolia*	*Polygraphus poligraphus*	**MN516732**	**MN517652**	**MN517679**	**MN517718**	**MN517752**
	39237 ^P^	136510	China	*Picea crassifolia*	*Polygraphus poligraphus*	**MN516733**	**MN517653**	**MN517680**	**MN517719**	**MN517753**
	39238		China	*Picea crassifolia*	*Polygraphus poligraphus*	**MN516734**	**MN517654**	**MN517681**	**MN517720**	**MN517754**

^1^**Bold** type = new species in the present study. ^2^CMW = Culture Collection of the Forestry and Agricultural Biotechnology Institute (FABI), University of Pretoria, Pretoria, South Africa; CBS = Westerdijk Fungal Biodiversity Institute, Utrecht, The Netherlands. ^H^ = ex-holotype; ^E^ = ex-epitype; ^P^ = ex-paratype ^3^ITS2 = the internal transcribed spacer 2 region of the nuclear ribosomal DNA gene; LSU = the 28S large subunit of the nrDNA gene; ACT= Actin; TUB = Beta-tubulin; CAL = Calmodulin; TEF-1α = Translation elongation factor 1-alpha; **Bold** type = Genbank accession numbers of sequences obtained in the present study.

### DNA extraction, PCR and sequencing

DNA extractions were done as described by Yin et al. (2015). For sequencing and phylogenetic analyses, five loci were amplified: internal transcribed spacer 2 and large subunit (ITS2-LSU), actin (ACT), beta tubulin (TUB), calmodulin (CAL) and translation elongation factor-1 alpha (TEF-1α). Primers used were: ITS3 and LR3 (White et al. 1990) for ITS2-LSU, Lepact-F and Lepact-R (Lim et al. 2004) for ACT, T10 (O’Donnell and Cigelnik 1997) and Bt2b (Glass and Donaldson 1995) for TUB, CL2F and CL2R (Duong et al. 2012) for CAL, EF2-F (Marincowitz et al. 2015) and EF2-R (Jacobs et al. 2004) for TEF-1α.

PCR reactions were conducted in 25 μL reaction mixtures containing 5 μL of Mytaq buffer (including MgCl_2_, dNTPs and reaction buffer), 0.5 μL of Mytaq polymerase (Bioline, USA), 0.5 μL of each primer (10 μM), and 16.5 μL of PCR grade water. PCR conditions for these five gene regions followed the protocols described by Yin et al. (2015). PCR products were purified with Sephadex G-50 columns (6%).

PCR products were sequenced with the same primers used for PCR, together with the Big Dye Terminator 3.1 cycle sequencing premix kit (Applied Biosystems, Foster City, California, USA). BigDye PCRs were conducted in 12 μL: sequencing Buffer 4.0 µL, Big Dye 1.0 µL, PCR Grade Water 4.0 µL, primer 1.0 µL, PCR product 2.0 µL; PCR conditions were: 1 min at 96 °C; 25 cycles of 10 sec at 96 °C, 5 sec at 50 °C, and 1min at 60 °C; and finally held at 12 °C. BigDye PCR products were also cleaned up with Sephadex. Sequence analyses were done on the ABI PRISM 3100 Genetic Analyzer (Applied Biosystems, Foster City, California, USA). Consensus sequences were generated from forward and reverse sequences in the CLC Main Workbench 6.0 (CLC Bio, Aarhus, Denmark).

### Phylogenetic analyses

Five sequence datasets were analyzed. The ITS2-LSU sequences of the ex-type isolate of every species in the *L. olivaceum* complex (Table 1) were compared with sequences of other known species in *Leptographium* obtained from GenBank to show the placement of the complex within the genus. Sequences of *Fragosphaeria purpurea* and *F. reniformis* were used to represent the outgroup taxa. Four protein coding gene regions (ACT, TUB, CAL, and TEF-1α) were sequenced (Table 1) for 39 isolates (Table 1) in order to delineate closely related species in the *L. olivaceum* complex. Sequences for *L. procerum* and *L. profanum* from the study of Yin et al. (2015) were selected to represent the outgroup taxa for the four protein-coding gene regions as well as in the combined dataset.

Alignments of loci were conducted in MAFFT 7.0 online (Katoh and Standley 2013), then checked manually in MEGA X (Kumar et al. 2018) and compared with the gene maps (Yin et al. 2015) to ensure that introns and exons were aligned appropriately. Three methods were used for phylogenetic analyses including Maximum parsimony (MP), Maximum Likelihood (ML), and Bayesian Inference (BI). A partition homogeneity test was conducted in PAUP* 4.0b10 (Swofford 2002) to consider the congruence of the four protein-coding gene regions before analyses of the combined dataset. The most important parameters used in phylogenetic analyses and statistical values related to all datasets analyzed are presented in Table 2.

MP analyses were executed in PAUP* 4.0b10 (Swofford 2002) with heuristic searches of 1000 replicates and tree bisection and reconnection (TBR) branch swapping options. Gaps were treated as the fifth base. Bootstrap analysis (1000 pseudo replicates) was performed to determine the confidence levels of the branch nodes. Tree length (TL), consistency Index (CI), retention Index (RI), Homoplasy Index (HI), and Rescaled Consistency Index (RC) were recorded after generating the trees.

The best substitution models (Table 2) for the two likelihood methods (ML and BI analyses) were selected congruously in jModelTest 2.1.1 (Pasoda 2008). MEGA X (Kumar et al. 2018) was used for ML analyses with Nearest-Neighbor-Interchange (NNI) branch swapping option. Node support values were determined using analysis of 1000 bootstrap pseudo replicates.

**Table 2.** Parameters used and statistical values related to all phylogenetic analyses in the present study.

		ITS2-LSU	ACT	βT	CAL	TEF-1α	Combined
Alignments	Number of taxa	59	41	41	41	41	41
	Total	603	809	288	579	781	2457
	Constant	456	622	209	435	479	1785
	Uninformative	46	20	8	22	45	95
	Informative	101	127	71	122	257	577
MP	Number of trees	396	13	4	15	10	12
	Tree length	289	276	154	404	619	1486
	CI	0.740	0.812	0.786	0.884	0.837	0.821
	RI	0.934	0.935	0.933	0.956	0.941	0.935
	RC	0.691	0.759	0.733	0.845	0.787	0.767
	HI	0.259	0.188	0.214	0.116	0.163	0.179
Model tests	Selected Models	GTR+I+G	HKY+I+G	HKY+G	HKY+I	HKY+G	HKY+I +G
ML	P-inv	0.378	0.527	–	0.623	–	0.441
	Gamma	0.257	0.287	0.179	–	0.618	0.712
BI	Burn-in	100	300	300	300	300	300

MP = maximum parsimony, ML = maximum likelihood, BI = Bayesian inference, Uninformative = Number of parsimony-uninformative characters, Informative = Number of parsimony-informative characters, CI = consistency index, RI = retention index, RC = rescaled consistency index, HI = homoplasy index, Subst. model = substitution models used in phylogenetic analyses, P-inv = proportion of invariable sites, Gamma = Gamma distribution shape parameter.

For BI analyses, the Markov Chain Monte Carlo (MCMC) method was used in MrBayes 3.2 (Ronquist et al. 2012). Four MCMC chains were simultaneously run from a random starting tree for five million generations. Trees were sampled every 100 generations. Burn-in values were determined in Tracer v1.7 (Rambaut et al. 2018). Trees sampled in the burn-in phase were discarded and posterior probabilities were calculated from all the remaining trees.

### Morphology and growth studies

In order to describe their morphology, isolates of new species were inoculated on to 2% water agar (WA, 20 g Difco agar and 1000 ml deionized water) amended with sterilized pine twigs (*Pinus pinaster*) and examined microscopically as described by Yin et al. (2015). Culture characteristics were recorded on Oatmeal agar (OA, 30 g oatmeal, 20 g Difco Bacto malt extract, from Becton, Dickinson and Company, and 1000ml deionized water) incubated at 25 °C for 10–14 days. Color descriptions were defined using the charts of Rayner (1970). Growth studies were conducted on 2 % Malt extract agar (MEA) following the procedure described by Yin et al. (2015).

## Results

### Phylogenetic analyses

**Figure 1.**
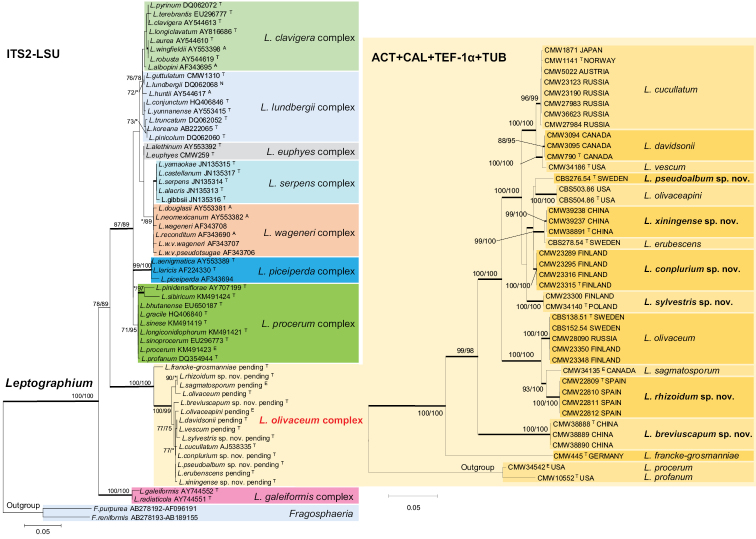
Left side: ML tree of the genus *Leptographium* generated from the ITS2-LSU DNA sequence data. Sequences generated from this study are printed in bold type. Bold branches indicate posterior probabilities values ≥0.95. Bootstrap values ≥75% are recorded at nodes as ML/MP. * Bootstrap values <75%. Scale bar represents 5 nucleotide substitutions per 100 nucleotides. Right side: ML trees of the *L. olivaceum* complex generated from the DNA sequences of combined four protein-coding gene regions, including ACT, CAL, TEF-1α, and TUB. Bold branches indicate posterior probabilities values ≥0.95. Bootstrap values ≥75% are recorded at nodes as ML/MP. * Bootstrap values <75%. Scale bar represents 5 nucleotide substitutions per 100 nucleotides.

The phylogenetic trees arising from the analyses of the ITS2-LSU data for *Leptographium* s.l. showed the *L. olivaceum* complex grouping between the *L. galeiformis* and *L. procerum* complexes with strong statistical support (Fig. 1). Within the complex, the ITS2-LSU sequences could not distinguish between some of the species, e.g. between *L. rhizoidum* and *L. sagmatosporum*; *L. davidsonii* and *L. vescum*; *L. conplurium*, *L. pseudoalbum* and *L. erubescens*. *Leptographium francke-grosmanniae* grouped peripheral to other species in the complex, but remained part of a strongly supported lineage including all the species under consideration.

**Figure 2.**
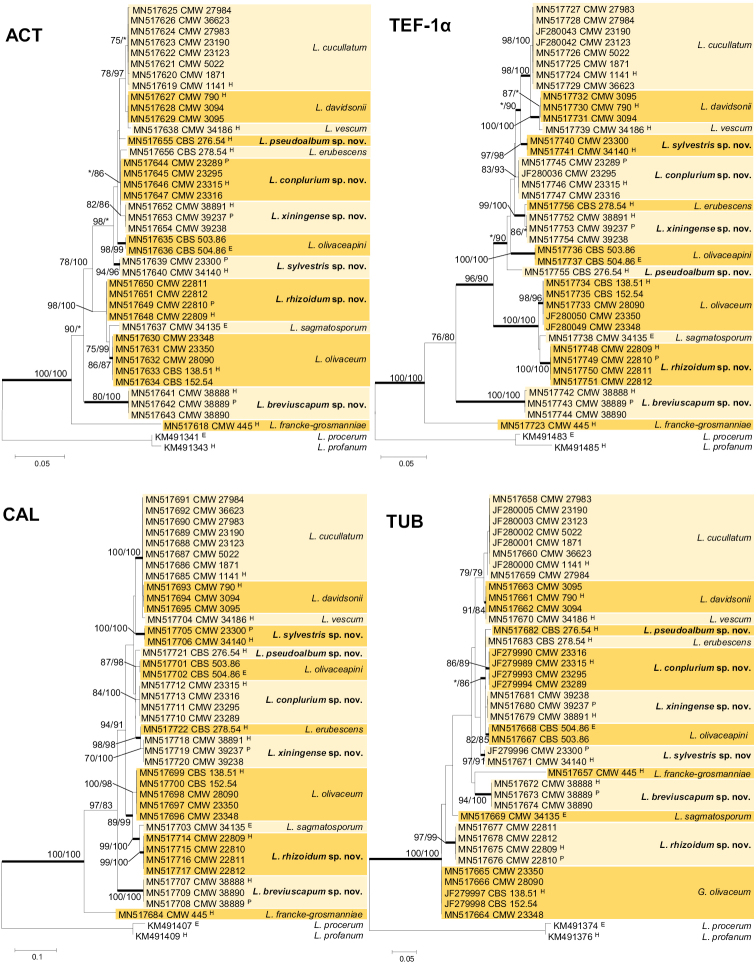
ML trees of the *L. olivaceum* complex generated from DNA sequences of four protein-coding gene regions. Bold branches indicate posterior probabilities values ≥0.95. Bootstrap values ≥75% are recorded at nodes as ML/MP. * Bootstrap values <75%. Scale bar represents nucleotide substitutions.

The ACT data matrix included part of exon 5 (sites 1–678), intron 5 (sites 679–785) and part of exon 6 (sites 786–809). The intron/exon composition of this gene region was congruent with that of the *L. procerum* complexes (Yin et al. 2015). Analyses of this gene region (Fig. 2) separated all known species and revealed six new taxa in the complex.

The TUB dataset included part of exon 4 (sites 1–41), intron 4 (sites 42–113), exon 5 (114–168) and part of exon 6 (sites 169–288). Intron 5 was lacking in the *L. olivaceum* complex, corresponding with most other species complexes in *Leptographium* s.l. (De Beer and Wingfield 2013). In the resulting phylogenies (Fig. 2), most known species and all new taxa could be separated, apart from the *L. davidsonii* and *L. vescum* isolates that formed a single clade.

The aligned DNA sequences for the CAL gene region included exon 3 (sites 1–16), intron 3 (sites 17–165), exon 4 (sites 166–291), intron 4 (sites 292–451), exon 5 (452–526), and part of exon 6 (sites 527–579). The intron/exon arrangement corresponded with that of the *L. clavigerum* and *L. procerum* complexes (Yin et al. 2015), with intron 5 lacking in this complex. Phylogenetic analyses of the CAL dataset (Fig. 2) recovered all currently accepted species in the complex.

The TEF-1α gene region used in phylogenetic analyses, included part of exon 3 (sites 1–9), intron 3 (sites 10–461), exon 4 (462–599), intron 4 (600–686), and part of exon 5 (687–781). Intron 4 of the TEF-1α gene was present in the *L. olivaceum* complex as is also true for the *L. procerum*, *L. galeiformis*, *L. wageneri* and *L. serpens* complexes, while it is absent in several other species complexes in *Leptographium* s. l. (De Beer and Wingfield 2013, Yin et al. 2015). Analysis of the TEF-1α dataset (Fig. 2) made it possible to separate all species in the complex.

The partition homogeneity test conducted on the combined data set for the four protein coding genes (ACT, TUB, CAL and TEF-1α) resulted in a P-value of 0.081, indicating that these regions could be combined. The MP, ML, and BI analyses generated were consistent with each other. Fourteen species with significant statistical support were defined in the *L. olivaceum* complex (Fig. 1), including eight known species (*L. cucullatum*, *L. davidsonii*, *L. vescum*, *L. olivaceapini*, *L. erubescens*, *L. olivaceum*, *L. sagmatosporum*, and *L. francke-grosmanniae*) and six new species from Europe and China.

### Morphology and growth studies

Isolates of the six new species emerging from this study were similar in growth in culture, with colors initially hyaline, later turning pale yellowish or pale olivaceous. Mononematous synnemata were common in the cultures and hyphae were superficial on the agar. The droplets containing conidia were initially hyaline, becoming yellowish with age. Morphological differences among all these new species are discussed in the *Notes* sections provided with the new species descriptions in the Taxonomy section. A sexual state was induced only in isolates of *L. sylvestris* after incubation at 25 °C for three weeks.

Other than *L. sylvestris* that grew fastest at 30 °C, the optimal growth temperature for all isolates of the new species was 25 °C. None of the isolates of the new species grew at 5 °C or 35 °C, only *L. rhizoidum* was able to grow (2.5 mm/d) at 35 °C.

### Taxonomy

Sequence data for 39 isolates included in the present study revealed 14 taxa in the *L. olivaceum* complex. One of these species, *L. erubescens*, was previously treated as a synonym of *L. cucullatum* but our data distinguished clearly between the two species. A new combination is thus provided for *L. erubescens*. Lectotypes and epitypes are designated here for *L. olivaceum*, *L. sagmatosporum* and *L. erubescens*. The remaining six taxa in the complex represented novel species and descriptions are provided for them.

#### Leptographium breviuscapum

Taxon classificationFungiOphiostomatalesOphiostomataceae

M.L. Yin, Z.W. de Beer & M.J. Wingf. sp. nov.

06018524-9DDB-5459-9CF5-1A1BFB961A6B

823576

Fig. 3

##### Etymology.

The epithet (brevius-, short, and -scapum, branch) refers to very short conidiophores.

##### Type.

CHINA, Qinghai province, from *Picea crassifolia* infested with *Polygraphus poligraphus*, Aug. 2010, *M.L. Yin* & *X.D. Zhou*, (PREM 60914 **holotype**, ex-holotype cultures CBS 136507 = CMW 38888); Qinghai province, from *Picea crassifolia* infested with *P. poligraphus*, Aug. 2010, *M.L. Yin & X.D. Zhou*, (PREM 60915 **paratype**, ex-paratype cultures: CBS 136508 = CMW 38889).

##### Description.

*Sexual state* not observed. *Conidiophores* occasionally observed on wood of WA, macronematous, synnematous, short, wide at the stipe, light brown to yellowish, expanding branches at the apex, 150–230 μm in length including conidiogenous apparatus, 20–25 μm wide at base, 40–45 μm wide at apex, 100–150 μm wide at conidiogenous apparatus. *Conidiogenous cells* discrete, hyaline, cylindrical, percurrent proliferation, (8–)9–13(–15) × 1.8–2.5 μm. *Conidia* hyaline, one-celled, smooth, ellipsoidal, (3.7–)4–4.5(–5) × 2.5–3 μm. *Culture characteristics*: Colonies on OA, hyaline at first, later becoming light yellowish in the center, mycelium superficial on agar. Mostly mycelium observed in culture, synnemata sparse. Optimal temperature for growth 25 °C, growth reduced at 10 °C and 30 °C, no growth at 35 °C.

**Figure 3.**
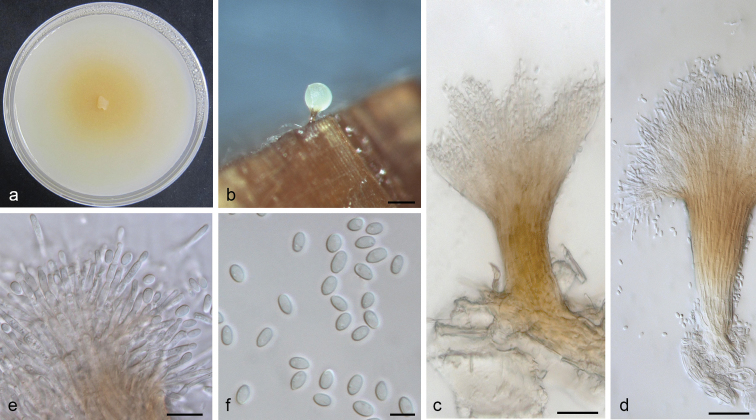
*Leptographium breviuscapum* sp. nov. (CMW 38888) **a** fourteen-days old culture on OA with black background **b** synnematous asexual state on wood tissue on WA**c–d** conidiophore **e** conidiogenous cells **f** conidia. Scale bars: 100 μm (**b**), 25 μm (**c**), 25 μm (**d**), 10 μm (**e**), 5 μm (**f**).

##### Host tree.

*Picea crassifolia*.

##### Insect vector.

*Polygraphus poligraphus*.

##### Distribution.

Qinghai, China.

*Note*: The asexual state of *L. breviuscapum* has very short conidiophores making it very easy to distinguish from that of other species in the complex.

##### Additional material examined.

Qinghai province, from *Picea crassifolia* infested with *Polygraphus poligraphus*, Aug. 2010, *M.L. Yin* & *X.D. Zhou*, (culture: CMW 38890). Yunnan province, from *Pinus yunnanensis* infested with *Tomicus yunnanense*, Sep. 2017, *M.L. Yin*, (culture: SCAU-475). Yunnan province, from *Pinus yunnanensis* infested with *Tomicus yunnanense*, Sep. 2017, *M.L. Yin*, (culture: SCAU-478).

#### Leptographium conplurium

Taxon classificationFungiOphiostomatalesOphiostomataceae

M.L. Yin, Z.W. de Beer & M.J. Wingf. sp. nov.

0D08C846-A29F-5447-AFD6-7AA0D062CE28

823572

Fig. 4

##### Etymology.

The epithet refers to synnemata produced abundantly in culture.

##### Type.

FINLAND, Ilomantsi, from *Picea abies* infested with *Dryocoetes autographus*, Aug. 2005, *Z.W. de Beer*, (PREM 60918-**holotype**, ex-holotype cultures: CBS 128923 = CMW 23315); Ilomantsi, from *P. abies* infested with *D. autographus*, Aug. 2005, *Z.W. de Bee*r, (PREM 60919-**paratype**, ex-paratype cultures: CBS 128834 = CMW 23289).

##### Description.

*Sexual state* not observed. *Conidiophores* macronematous, synnematous, 300–700 μm including conidiogenous apparatus, synnemata occasionally swollen at the base, frequently swollen at the stipe, brown to black, expanding branches at the apex, (25–)40–50(–80) μm in width, abundantly produced in culture. *Conidiogenous cells* discrete, terminal, hyaline, cylindrical, (8–)12–17(–20) × 1.5–2.3 μm. *Conidia* hyaline, one-celled, ellipsoidal to cylindrical, (3.9–)4.3–4.9(–6.3) × 1.9–2.5 μm. *Culture characteristics*: colonies on OA, hyaline at first, later becoming light yellowish in the center, concentric rings present, hyphae hyaline, appressed and immersed. Optimal growth temperature is 25 °C with radial growth rate 2.5 (± 0.5) mm/d, growth reduced at 10 °C and 30 °C, no growth at 35 °C.

**Figure 4.**
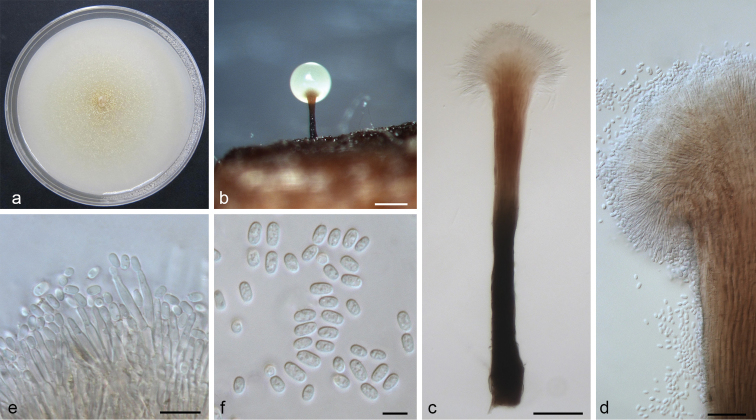
*Leptographium conplurium* sp. nov. (CMW 23315). **a** fourteen-days old culture on OA with black background; b. synnematous asexual state on wood tissue on WA**c** conidiophore **d** conidiogenous apparatus **e** conidiogenous cells **f** conidia. Scale bars: 200 μm (**b**), 50 μm (**c**), 20 μm (**d**), 10 μm (**e**), 5 μm (**f**).

##### Host tree.

*Picea abies*.

##### Insect vectors.

*Dryocoetes autographus*, *Hylastes brunneus*.

##### Distribution.

Finland.

##### Notes.

All isolates of this species were initially recognized as a cryptic species closely related to *L. cucullatum* and *L. olivaceapini* by Linnakoski et al. (2012). Our results confirmed that they represent an undescribed taxon.

##### Additional material examined.

FINLAND, Ilomantsi, from *Picea abies* infested with *Dryocoetes autographus*, Aug. 2005, *Z.W. de Beer*, (culture: CMW 23295); Ilomantsi, from *P. abies* infested with *Hylastes brunneus*, Aug. 2005, *Z.W. de Beer*, (culture: CMW 23316).

#### Leptographium cucullatum

Taxon classificationFungiOphiostomatalesOphiostomataceae

(H. Solheim) M.L. Yin, Z.W. de Beer & M.J. Wingf. comb. nov.

63322A10-8802-5AD9-BFE8-0C385FAC3096

831546

≡ Ophiostoma cucullatum H. Solheim, Nord. J. Bot. 6: 202 (1986). (Basionym)
≡ Grosmannia cucullata (H. Solheim) Zipfel, Z.W. de Beer & M.J. Wingf., Zipfel et al. (2006) Stud. Mycol. 55: 90.

##### Type.

NORWAY, Vestfold, Lardal, from *Ips typographus* caught when leaving a log of *Picea abies*, 20 Aug 1981, *H. Solheim*, (CBS H-15306 and CBS H-3560-**holotype**, ex-holotype cultures: CMW 1140 = CBS 218.83 = 81-83/16).

##### Descriptions.

Solheim (1986, pp 202–203, fig. 2); Wingfield et al. (1989, pp 92–95, figs 1–10); Yamaoka et al. (1997, pp 1220–1221 figs 22–26); Harrington et al. (2001, pp 128–129, figs 41, 44).

##### Host trees.

*Picea abies*, *Picea jezoensis*, *Pinus sylvestris*.

##### Insect vectors.

*Dryocoetus autographus*, *Ips typographus*, *Ips typographus japonicus*.

##### Distributions.

Europe (Austria, Norway, Poland, Russia), Japan

##### Notes.

Harrington et al. (2001) suggested that *Phialographium erubescens* represented the asexual state of *L. cucullatum*. Comprehensive data from the present study distinguish between the two species. See details under *L. erubescens*.

##### Additional material examined.

AUSTRIA, Tyrol, Ehrwald, from *I. typographus* in *Picea abies*, July 1997, *T. Kirisits*, CMW 5022; JAPAN, Hokkaido, Furano, from an adult of *Ips typographus japonicus* in *Picea jezoensis*, 31 July 1991, *Y. Yamaoka*, CMW 1871 = JCM 8816; RUSSIA, Ohtama, from *I. typographus* in *P. abies*, June 2004, *J. Ahtiainen*, CMW 23123 = CBS 128299;RUSSIA, Lisino-Corpus,from *I. typographus* in *Pinus sylvestris*, *R. Linnakoski*, CMW 23190; RUSSIA, Kivennapa, Lintula, from *Dryocoetus autographus* in *P. abies*, Oct 2007, *R. Linnakoski*, CMW 27983, CMW 27984; RUSSIA, Karelia, from *I. typographus* in *P. abies*, *H. Roininen*, CMW 36623.

#### Leptographium davidsonii

Taxon classificationFungiOphiostomatalesOphiostomataceae

(Olchow. & J. Reid) M.L. Yin, Z.W. de Beer & M.J. Wingf. comb. nov.

6DDD1B84-0353-55BC-AFBD-16B797429197

831547

≡ Ceratocystis davidsonii (Olchow. & J. Reid), Can. J. Bot. 52: 1698 (1974). (Basionym)
≡ Ophiostoma davidsonii (Olchow. & J. Reid) H. Solheim, Nord. J. Bot. 6: 203 (1986).
≡ Grosmannia davidsonii (Olchow. & J. Reid) Zipfel, Z.W. de Beer & M.J. Wingf., Zipfel et al., Stud. Mycol. 55: 90 (2006).

##### Type.

CANADA, British Columbia, Seymour Arm, from *Pseudotsuga menziesii*, 1971, *J. Reid*, (WIN (M) 71-30-**holotype**, ex-holotype cultures: CMW 790 = IMI 176524 = JCM 7867).

##### Descriptions.

Olchowecki & Reid (1974, pp 1698–1699, figs 230–238); Upadhyay (1981, pp 42–43, figs 58–62); Mouton et al. (1993, pp 376–377, figs 15–18); Ohtaka et al. (2002, pp 154–156, figs 6–10).

##### Host trees.

*Abies veitchii*, *Picea* sp, *Pseudotsuga menziesii*.

##### Insect vector.

*Dryocoetes hectographus*.

##### Distribution.

USA, Japan.

##### Notes.

The orange section shaped to hemispherical ascospores makes this species distinct from others in the complex (Ohtaka et al. 2002). This fungus was also reported associated with *Dryocoetes hectographus* on *Abies veitchii* in Japan based on morphology (Ohtaka et al. 2002), but the identity of the Japanese isolates needs to be verified with DNA sequences.

##### Additional material examined.

CANADA, British Columbia, Lake Louise, from small Scolytinae sp. in *Picea* sp. Aug 1994, *M. J. Wingfield*, (cultures: CMW 3094, CMW 3095).

#### Leptographium erubescens

Taxon classificationFungiOphiostomatalesOphiostomataceae

(Math.-Käärik) M.L. Yin, Z.W. de Beer & M.J. Wingf. comb. nov.

4895DF9B-55E1-5AA0-970A-4706D1CC08F1

823577

≡ Graphium erubescens Math.-Käärik, Medd. Skogs for skninginst. 43: 62 (1953). (Basionym)
≡ Pesotum erubescens (Math.-Käärik) G. Okada, Stud. Mycol. 45: 184 (2000).
≡ Phialographium erubescens (Math.-Käärik) T.C. Harr. & McNew, Mycologia 93: 129 (2001).

##### Type.

SWEDEN, from pine poles and board, *A. Mathiesen-Käärik*, **lectotype** designated here, represented by line drawings (fig. 8b, p. 58; fig. 9d–f, p. 61) from Mathiesen-Käärik (1953), **MBT 379456**; Uppland, Skutskär, from piled timber of *Pinus sylvestris*, 1952, *A. Mathiesen-Käärik*, (Isotype CBS H-7193, CBS H-7194, ex-type cultures: CMW 40672 = CBS 278.54 = JCM 9747 = No. Sk 13-52).

##### Descriptions.

Mathiesen-Käärik (1953, p.62, figs8b, 9f–d); Harrington et al. (2001, pp 128–129, figs 42, 43, 45).

##### Host tree.

*Pinus sylvestris*.

##### Insect vector.

unknown.

##### Distribution.

Sweden.

##### Notes.

This species was first described by Mathiesen-Käärik (1953) from pine timber in Sweden. No specimen numbers and very little detail (e.g. no host locality or collection dates) were provided in the protologue. Furthermore, no specimen number and little detail are listed under this species name in the herbarium of the Museum of Evolution, Uppsala, which incorporated Mathiesen-Käärik’s collection. However, in 1954 she deposited an isolate (No. Sk 13-52) in the CBS labeled as *L. erubescens*. Two dried specimens (CBS H-7193, CBS H-7194) are linked to this isolate and these are labeled as isotypes. It is reasonable to assume that this isolate represents the original material, but there is no conclusive evidence that this is true. We have thus designated the line drawings from the protologue (Mathiesen-Käärik 1953) as the lectotype.

Harrington et al., (2001) suggested that *Graphium erubescens* (as *Phialographium erubescens*) represented the asexual state of *L. cucullatum* (as *O. cucullatum*) based on ITS sequences. However, based on sequences produced in the present study, the ex-type culture of *L. erubescens* differs from that of *L. cucullatum* in 1bp in ITS2-LSU, 17 bp in ACT, 17 bp in BT, 30 bp in CAL, and 48 bp in TEF-1α. We have thus treated these species as distinct and have provided a new combination for *L. erubescens*.

#### Leptographium francke-grosmanniae

Taxon classificationFungiOphiostomatalesOphiostomataceae

(R.W. Davidson) K. Jacobs & M.J. Wingf., Leptographium species: p. 99 (2001)

1FF3D6C8-08D2-5A1E-B096-D75FEE40EDC0

MB375135

≡ Ceratocystis francke-grosmanniae R.W. Davidson, Mycologia 63: 6 (1971). (Basionym)
≡ Ophiostoma francke-grosmanniae (R.W. Davidson) De Hoog & R.J. Scheff., Mycologia 76: 297 (1984).
≡ Grosmannia francke-grosmanniae (R.W. Davidson) Zipfel, Z.W. de Beer & M.J. Wingf., Stud. Mycol. 55: 90 (2006).

##### Type.

GERMANY, Reinbeck near Hamburg, from *Quercus* sp. associated with *Hylecoetus dermestoides*, May 1967, *H. Francke-Grosmann*, (**holotype** BPI 595654, ex-holotype cultures: RWD 828 = ATCC 22061 = CBS 356.77 = CMW 445).

##### Descriptions.

Davidson (1971, pp 6–7, figs 1, 10, 11, 17); Upadhyay (1981, p. 45, figs 73–78); Mouton et al. (1992, figs 1–11); Wingfield (1993, p. 48, figs 6–7); Jacobs and Wingfield (2001, pp 99–102, figs 73–75).

##### Host tree.

*Quercus* sp.

##### Insect vector.

*Hylecoetus dermestoides*.

##### Distribution.

Germany.

##### Notes.

*Leptographium francke-grosmanniae* groups peripheral to other species in the *L. olivaceum* complex (Figs 1–3). Morphologically, the ascospores are almost cylindrical and its ascomatal necks correspond with other species in the complex. But *L. francke-grosmanniae* produces mononematous conidiophores, in contrast to the synnemata produced by the other species, which also explains why it is the only species in the complex previously treated in *Leptographium*. The mode of conidiogenesis of *L. francke-grosmanniae* (Mouton et al. 1992) appears similar to that of other species where the conidiogenous cells that appear phialidic under a light microscope arise from percurrent proliferation (Wingfield et al. 1989, Wingfield et al. 1991, Mouton et al. 1993). However, the apices of the apparent “phialides” are substantially more flared than those of other species in the complex and they could be more different than assumed by Mouton et al. (1993). *Leptographium francke-grosmanniae* is also unusual in the *L. olivaceum* complex in having an angiosperm host.

*Leptographium francke-grosmanniae* was originally described as *Ceratocystis francke-grosmanniae* from larval galleries of *Hylecoetus dermestoides* on *Quercus* sp. in Germany (Davidson 1971). De Beer and Wingfield (2013) showed that sequences for this species produced in different studies were inconsistent. Based on comparisons of the ITS2 region, the sequences of ex-holotype generated in the present study are consistent with those produced by Mullineux and Hausner (2009) for ATCC 22061 and Hamelin et al. (unpublished) for CBS 356.77, but differ substantially from sequences produced by Jacobs et al. (2001b). In the LSU gene region, our sequences are identical to those of Hausner et al. (2000), but they differed from that of Jacobs et al. (2001a, b) for CMW 445.In the β-tubulin gene region, the sequence of CMW 445 in the present study was consistent with that provided by Kim et al. (2004) for CMW 445 and Hamelin et al. (unpublished sequence in GenBank) for CBS 356.77. We thus suggest that the two sequences for *L. francke-grosmanniae* produced by Jacobs et al. (2001a, b) are incorrect. Sequences of another isolate from the USA (CMW 2975), previously identified as *L. francke-grosmanniae* (Zipfel et al. 2006), differ substantially from the ex-holotype culture. Thus, this isolate (CMW 2975) does not represent *L. francke-grosmanniae*, and its taxonomic placement needs reconsideration.

#### Leptographium olivaceum

Taxon classificationFungiOphiostomatalesOphiostomataceae

(Math.-Käärik) M.L. Yin, Z.W. de Beer & M.J. Wingf. comb. nov.

E3A8AA8E-0410-571E-8A85-E09D88971D1B

831548

≡ Ophiostoma olivaceum Math.-Käärik, Svensk. Bot. Tidskr. 45: 212 (1951). (Basionym)
≡ Ceratocystis olivacea (Math.-Käärik) J. Hunt, Lloydia 19: 29 (1956).
≡ Grosmannia olivacea (Math.-Käärik) Zipfel, Z.W. de Beer & M.J. Wingf., Zipfel et al., Stud. Mycol. 55: 91 (2006).

##### Type.

SWEDEN, Hällnäs, Västerbotten, from the galleries of *Acanthocinus aedilis* in pine wood, *A. Mathiesen-Käärik*, **lectotype** designated here, represented by line drawings (fig. 2a–g, p. 213) from Mathiesen-Käärik (1951), **MBT 379459**; from dead wood of *Pinus sylvestris*, Jan 1949, *A. Mathiesen-Käärik*, (ex-type cultures: CMW 31059 = CBS 138.51, MBT 2063).

##### Descriptions.

Mathiesen-Käärik (1950, p. 298); Mathiesen-Käärik (1951, pp 212–215, fig. 2); Hunt (1956, pp 29–30); Griffin (1968, pp 707–708, figs 49–52, 82); Olchowecki and Reid (1974, pp 1699–1700, Pl. XIII fig. 262); Upadhyay (1981, pp 52–54, figs 116–121); Mouton et al. (1993, pp 376–377, figs 19–22).

##### Host trees.

*Betula papyrifera*, *Picea abies*, *Picea mariana*, *Pinus sylvestris*.

##### Insect vectors.

*Acanthocinus aedilis*, *Dendroctonus rufipennis*, *Ips typographus*, *Polygraphus rufipennis*.

##### Distributions.

Canada, Finland, Russia, Sweden, USA.

##### Notes.

This species was first described invalidly (no Latin diagnosis) from *Pinus sylvestris* infested by a longhorn beetle *Acanthocinus aedilis* in Sweden (Mathiesen-Käärik 1950). Mathiesen-Käärik (1951) then validated the name with a more detailed description accompanied by a Latin diagnosis. In the original descriptions of *L. olivaceum* by Mathiesen-Käärik (1950, 1951), the host tree, beetle and location of the collection was noted, but no mention was made of a specimen. The herbarium specimens of Mathiesen-Käärik were initially curated in the herbarium of the Statens Skogsforsknings institut, Experimentalfältet, Sweden. The collection was later incorporated into the herbarium of the Museum of Evolution, Uppsala. Only one herbarium specimen (UPS:BOT:F-130986) of *L. olivaceum*, collected from the same host, beetle and location by T. Hedquist, is available from that collection. However, an isolate of *L. olivaceum* (No. 297-49 = CBS 138.51), collected in 1949, also from the original host and location, was deposited in the CBS by Mathiesen-Käärik in 1951. Although we were not able to confirm that this isolate was from the original collection, it was treated as the ex-type culture of the species in previous studies (Duong et al. 2012, Linnakoski et al. 2012, De Beer and Wingfield 2013). In view of the absence of concrete evidence that this isolate represents the original material, we have designated the line drawings from the protologue (Mathiesen-Käärik 1951) as lectotype.

More recently, it was reported from *Picea abies* and *Pinus sylvestris* infested by *Ips typographus* and *Dryocoetes autographus* in Finland and Russia, in a study where the identities were confirmed using DNA sequence analyses (Linnakoski et al. 2012). Griffin (1968) reduced *L. vescum* to synonymy with *L. olivaceum*, but data from the present study confirmed that these two species are phylogenetically distinct.

##### Additional material examined.

FINLAND, Jouhteninen, from *Ips typographus* in *Picea abies*, July 2005, *Z.W. de Beer*, (cultures: CMW 23348 = CBS 128836, CMW 23350 = CBS 128837). RUSSIA, Uuksujärvi, from *I. typographus* in *Pinus sylvestris*, Oct 2007, *R. Linnakoski*, (culture CMW 28090). SWEDEN, Oct 1954, *A. Mathiesen-Käärik*, (cultures: CMW 31060 = CBS 152.54).

#### Leptographium olivaceapini

Taxon classificationFungiOphiostomatalesOphiostomataceae

(R.W. Davidson) M.L. Yin, Z.W. de Beer & M.J. Wingf. comb. nov.

AC704DF1-9357-5B49-8AAA-DAED89C4592D

831549

≡ Ceratocystis olivaceapini R.W. Davidson, Mycologia 63: 7 (1971). (Basionym)
≡ Ophiostoma olivaceapini (R.W. Davidson) K.A. Seifert & G. Okada, *In* Okada et al., Can. J. Bot. 76: 1504 (1998).
≡ Grosmannia olivaceapini (R.W. Davidson) Z.W. de Beer, R. Linnakoski & M.J. Wingf., *In* Linnakoski et al., Antonie van Leeuwenhoek 102: 375–399 (2012).

##### Type.

USA, New Mexico, Santa Fe, from *Pinus ponderosa* tree infested *Dendroctonus* sp. and other bark beetles, 10 July 1964, *R.W. Davidson*, (**holotype** BPI 595910 = RWD 548D; BPI 595914 = RWD 548D isotype); USA, Arizona, Flagstaff, from *Pinus ponderosa* infested with *Dendroctonus* sp., 24 July 1964, *R.W. Davidson*, (BPI 596223= RWD 581-D isotype); Arizona, Flagstaff, from *P. ponderosa* infested with *Dendroctonus* sp., 3 Oct 1986, *T. Hinds*, (**epitype**PREM 61051, designated here, ex-epitype cultures CBS 504.86 = CMW 116 = COLO 479, **MBT 379458**).

##### Descriptions.

Davidson (1971, pp 7–10, figs 2, 12, 18); Upadhyay (1981, p. 54, figs 122–129); Mouton et al. (1993, pp 372–373, figs 1–4).

##### Host trees.

*Pinus ponderosa*.

##### Insect vectors.

*Dendroctonus* sp.

##### Distribution.

USA.

##### Notes.

No living culture associated with the holotype (BPI 595910) or isotype (BPI 595914) of *L. olivaceapini* exists. However, T. Hinds, a collaborator of R.W. Davidson and later curator of the RWD culture collection, provided an isolate (COLO 479) labeled as *C. olivaceapini* to M.J. Wingfield, who later deposited this in the CBS (CBS 504.86). The species name and origin provided by Hinds with the isolate corresponds to a second specimen mentioned by Davidson (1971, p. 10) in the protologue (RWD 581-D = BPI 596223). In our opinion, the isolate (COLO 479) most probably originated from the specimen (RWD 581-D). We could not confirm with certainty that BPI 296223 originated from RWD 581-D and thus designated a dried culture of COLO 479 as the epitype for *L. olivaceapini*.

*Additional Material examined*: USA, Arizona, Flagstaff, from *P. ponderosa* infested with *Dendroctonus* sp., 3 Oct 1986, *T. Hinds*, (PREM 61051, cultures CBS 504.86 = CMW 116 = COLO 479). Minnesota, *Pinus resinosa*, Nov 1986, M.J. Wingfield, (cultures: CBS 503.86 = CMW 63).

#### Leptographium pseudoalbum

Taxon classificationFungiOphiostomatalesOphiostomataceae

M.L. Yin, Z.W. de Beer and M.J. Wingf. sp. nov.

4ECD53D1-B9F3-5404-A478-7EABEC9BF2FB

823571

Fig. 5

##### Etymology.

The epithet refers to the previous, incorrect identification of the ex-holotype isolate of this species as *Graphium album*.

##### Type.

SWEDEN, from *Pinus sylvestris* infested by *Tomicus piniperda*, 1953, *Mathiesen-Käärik*, (PREM 61050-**holotype**, ex-holotype cultures: CBS 276.54 = CMW 40671 = JCMW 9774 = C 1225).

##### Description.

*Sexual state* not observed. *Conidiophores* macronematous, synnematous, 120–270 μm including conidiogenous apparatus, synnemata frequently swollen at base, frequently wider at stipe, expanding branches at apex, brown to hyaline, (11–)25–34(–40) μm in width. *Conidiogenous cells* discrete, terminal, percurrent and phialidic proliferation, hyaline, cylindrical, (9–)10–14(–18) × 1.8–2.8 μm. *Conidia* hyaline, one-celled, ellipsoidal to cylindrical, (3.5–)4.3–5.2(–6.5) × 2.4–3.3 μm. *Cultural characteristics*: Colonies on OA, hyaline at first, later becoming white and gray in the center, hyphae hyaline, appressed and immersed, aerial mycelium frequently present on wood tissue, phialographium-like asexual morph abundant. Optimal growth temperature on MEA:25 °C with radial growth rate 3.0 (± 0.5) mm/d, while growth slightly reduced at 10 °C and 30 °C, and no growth occurred at 35 °C.

**Figure 5.**
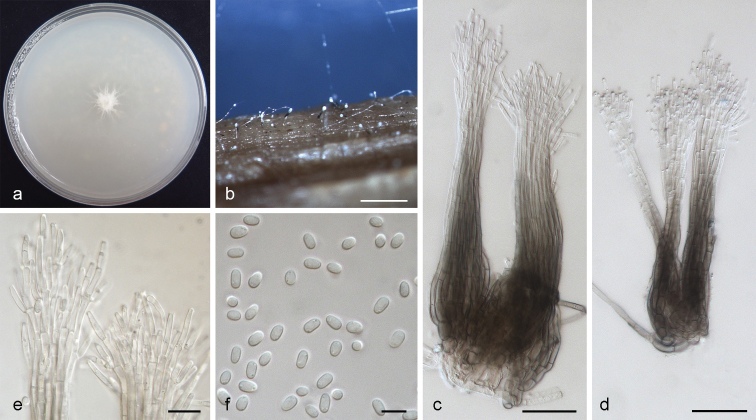
*Leptographium pseudoalbum* sp. nov. (CBS 276.54) **a** fourteen-days old culture on OA with black background; b. synnematous asexual state on wood tissue on WA**c–d** conidiophore **e** conidiogenous cells **f** conidia. Scale bars: 200 μm (**b**), 25 μm (**c**), 25 μm (**d**), 10 μm (**e**), 5 μm (**f**).

##### Host.

*Pinus sylvestris*.

##### Insect vector.

*Tomicus piniperda*.

##### Distribution.

Sweden.

##### Notes.

This species was initially identified as *Graphium album* (Corda) Sacc. by Mathiesen-Käärik (1953). However, Okada et al. (2000) and Harrington et al. (2001) questioned the identification by Mathiesen-Käärik (1953) and showed that this isolate belonged in the Ophiostomatales and grouped close to *L. erubescens*. This study showed that Mathiesen-Käärik’s isolate representing an undescribed species in the *L. olivaceum* complex, for which we have provided the name *L. pseudoalbum*.

#### Leptographium rhizoidum

Taxon classificationFungiOphiostomatalesOphiostomataceae

M.L. Yin, Z.W. de Beer and M.J. Wingf. sp. nov.

D9A34037-97F6-5AD7-87CE-CAA88E1293B7

823575

Fig. 6

##### Etymology.

The epithet refers to the rhizoid-like structures at the synnematal bases.

##### Type.

SPAIN, Morga, from *Pinus radiata* infested by *Hylastes ater*, July. 2004, *P. Romon & X.D. Zhou*, (PREM 60922-**holotype**, ex-holotype cultures: CBS 136512 = CMW 22809); Morga, from *Pinus radiata* infested by *Hylastes attenuatus*, July. 2004, *P. Romon & X.D. Zhou*, (PREM 60923-**paratype**, ex-paratype cultures: CBS 136513 = CMW 22810).

##### Description.

*Sexual state* not observed. *Conidiophores* macronematous, synnematous, 200–350 μm including conidiogenous apparatus, synnemata frequently swollen at the base, frequently wider at the stipe, brown to light brown, expanding branches at the apex, (15–)35–45(–70) μm in width. *Conidiogenous cells* discrete, terminal, percurrent and phialidic proliferation, hyaline, cylindrical,(10–)14–17(–19) × 2–3 μm. *Conidia* hyaline, one-celled, cylindrical to obovoid, (5.1–)6.5–7.8(–10.5) × 2.1–3.5 μm. *Cultural characteristics*: Colonies on OA, hyaline at first, later becoming olivaceous in the center, hyphae hyaline, appressed and immersed, aerial mycelium frequently present on wood tissue, synnemata abundant in WA cultures, Optimal growth temperature on MEA is 25 °C with radial growth rate 6.0 (± 0.5) mm/d, growth slightly reduced at 10 °C and 35 °C.

**Figure 6.**
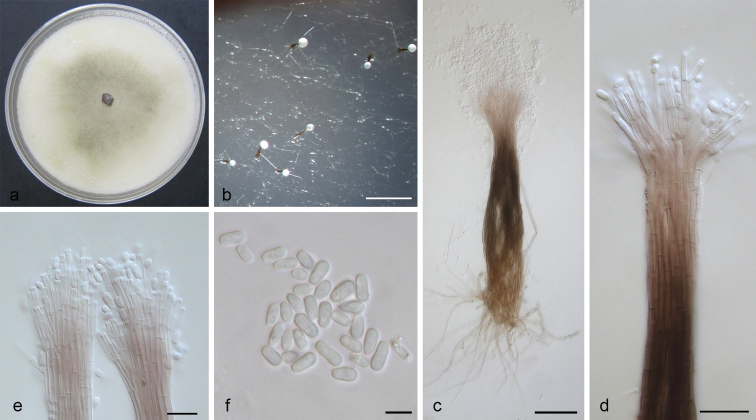
*Leptographium rhizoidum* sp. nov. (CMW 22809). **a** fourteen-days old culture on OA with black background **b** synnematous asexual state on wood tissue on WA**c** conidiophore **d** conidiogenous apparatus **e** conidiogenous cells **f** conidia. Scale bars: 200 μm (**b**), 50 μm (**c**), 20 μm (**d**), 10 μm (**e**), 5 μm (**f**).

##### Host tree.

*Pinus radiata*.

##### Insect vectors.

*Hylastes ater*, *H. attenuatus*, *Hylurgops palliatus*, *Ips sexdentatus*.

##### Distribution.

Spain.

*Note*: Isolates of *L. rhizoidum* from pine-infesting bark beetles in Spain were initially identified as *L. olivaceum* based on ITS sequences by Romon et al. (2007). Our data showed them to be distinct from that species. This species produced more abundant and longer rhizoids than others in the complex.

*Other Material examined*: SPAIN, Morga, from *Pinus radiata* infested by *Ips sexdentatus*, July. 2004, *P. Romon & X.D. Zhou*, (culture: CMW 22811); Morga, from *P. radiata* infested by *Hylurgops palliatus*, July. 2004, *P. Romon & X.D. Zhou*, (culture: CMW 22812).

#### Leptographium sagmatosporum

Taxon classificationFungiOphiostomatalesOphiostomataceae

(E.F. Wright & Cain) M.L. Yin, Z.W. de Beer & M.J. Wingf. comb. nov.

88DA1577-F14D-5B3E-86B5-EDA29C32EDA5

831550

≡ Ceratocystis sagmatospora E.F. Wright & Cain, Can. J. Bot. 39: 1226 (1961). (Basionym).
≡ Phialographium sagmatosporae H.P. Upadhyay and W.B. Kendr., Mycologia 66: 183 (1974).
≡ Ophiostoma sagmatosporum (E.F. Wright & Cain) H. Solheim, Nord. J. Bot. 6: 203 (1986).
≡ Graphium sagmatosporae (H.P. Upadhyay & W.B. Kendr.) M.J. Wingf. & W.B. Kendr., Mycol. Res. 95: 1332 (1991).
≡ Pesotum sagmatosporum (H.P. Upadhyay & W.B. Kendr.) G. Okada & K.A. Seifert, in Okada et al., Can. J. Bot. 76: 1504 (1998).
≡ Grosmannia sagmatospora (E.F. Wright & Cain) Zipfel, Z.W. de Beer & M.J. Wingf., *In* Zipfel et al. Stud. Mycol. 55: 91 (2006).

##### Type.

CANADA, Ontario, Ontario, NE. of Mansfield, Dufferin Co., from *Pinus resinosa*, Nov. 8 1958, *E.F. Wright* &*R.F. Cain*, **lectotype** designated here, represented by line drawings (fig. 23, p. 1225, figs 24–33, p. 1228) from Wright and Cain (1961), **MBT 379455**; Ontario, Stittsville, 13 Lucas Lane, 4511.9 N 7558.8 W, from old bark beetle galleries in *Pinus strobus*, Sept. 2000, *K. Jacobs*, (**epitype**PREM 61054, designated here, ex-epitype cultures: CMW 34135 = CBS 113452, **MBT 379454**).

##### Descriptions.

Wright and Cain (1961, pp 1226–1229, figs 23–33); Griffin (1968, pp 708, 712–713); Olchowecki and Reid (1974, p. 1701, Pl. XIII figs 254, 257); Upadhyay (1981, p. 60, figs 167–171).

##### Host trees.

*Pinus strobus*, *Picea mariana*.

##### Insect vectors.

unknown bark beetle species.

##### Distribution.

Canada.

##### Notes.

This species was originally described from bark beetle galleries and freshly cut surfaces of *Picea mariana*, *Pinus resinosa* and *Pinus strobus* in Canada (Wright and Cain 1961). The Royal Ontario Museum Fungarium (TRTC), Canada, informed the authors of this study that the holotype (TRTC 36427) of *L. sagmatosporum* was permanently lost. There is also no living culture available from the holotype. We have thus designated the line drawings in the protologue as the lectotype. An isolate (CMW 34135), also from pine in Ontario, identified as *L. sagmatosporum* based on morphology (K. Jacobs, unpublished) and used in previous studies to represent the species (Duong et al. 2012, Linnakoski et al. 2012, De Beer and Wingfield 2013), its dry specimen is designated here as the epitype.

*Additional Material examined*: CANADA, Ontario, NE. of Mansfield, Dufferin Co., from *Pinus resinosa*, Nov. 8 1958, *E.F. Wright* & *R.F. Cain*, TRTC 34600; NW. of Nobleton, York Co., from *Pinus strobus*, July 1 1957, *E.F. Wright* & *R.F. Cain*, TRTC 33034; Twp. West of 11 H, Challener Lake, Sudbury Dist., from *Pinus strobus*, June 20 1960, *E.F. Wright* & *R.F. Cain*, TRTC 36245, 36251, 36255, 36264, 36265;Twp. 5F, Aubinadong R., Algoma Dist, from *Pinus strobus*, June 17 1960, *E.F. Wright* & *R.F. Cain*, TRTC 36246; Twp. West of 11 H, Challener Lake, Sudbury Dist., from *Picea mariana*, June 20 1960, *E.F. Wright* & *R.F. Cain*, TRTC 36263.

#### Leptographium sylvestris

Taxon classificationFungiOphiostomatalesOphiostomataceae

M.L. Yin, Z.W. de Beer and M.J. Wingf. sp. nov.

B4B99C40-4783-5175-B8C8-81DD0CF31211

823574

Fig. 7

##### Etymology.

The epithet refers to the host species where the holotype was collected.

##### Type.

POLAND, Chrosnica, from *Pinus sylvestris*, Jan. 2008, *R. Jankowiak*, (PREM 60920-**holotype**, ex-holotype cultures: CBS 136511 = CMW 34140). FINLAND, Jouhteninen, from *Picea abies* infested with *Ips typographus*, Aug. 2005, *Z.W. de Beer*, (PREM 60921-**paratype**, ex-paratype cultures: CBS 128833 = CMW 23300).

##### Description.

*Sexual state* develop on wood on WA in 14–21 days. *Perithecia* superficial on wood and agar, base brown to black, globose, unornamented, 91–110 μm in diameter, necks dark brown, cylindrical, slightly curved, 200–480 μm long (including ostiolar hyphae), 26–32 μm wide at base, 15–21 μm wide at the tip. *Ostiolar hyphae* present, pale brown, straight, septate, numerous, divergent, tapering at the tip, up to 190 μm long. *Asci* not seen. *Ascospores* one-celled, hyaline, fusiform to orange section shaped in side view, ellipsoidal in face view, globose in end view, (4.0–)4.5–5.5(–5.8) × (2.5–)2.8–3.7(–3.9) μm including hyaline gelatinous sheath, 0.3–0.6 μm thick. *Conidiophores* macronematous, synnematous, swollen at the base, occasionally wider at the stipe, brown to light brown, expanding branches at the apex, 260–500 × 14–57 μm including conidiogenous apparatus. *Conidiogenous cells* discrete, hyaline, cylindrical, 2–3 per branch, percurrent proliferation, (10–)11–15(–18) × 1.5–2.5 μm. *Conidia* hyaline, obovate to clavate, (3.6–)4.5–4.9(–5.2) × (1.6–)1.7–1.9(–2.1) μm. *Cultural characteristics*: Colonies on OA, hyaline at first, later becoming dark yellowish in the center, mycelium appressed and immersed, Perithecia and *Pesotum*-like asexual morph co-occur in culture. Optimal growth temperature is 30 °C, radial growth rate 5.0 (± 0.5) mm/d, growth reduced at 10 °C, no growth at 35 °C.

**Figure 7.**
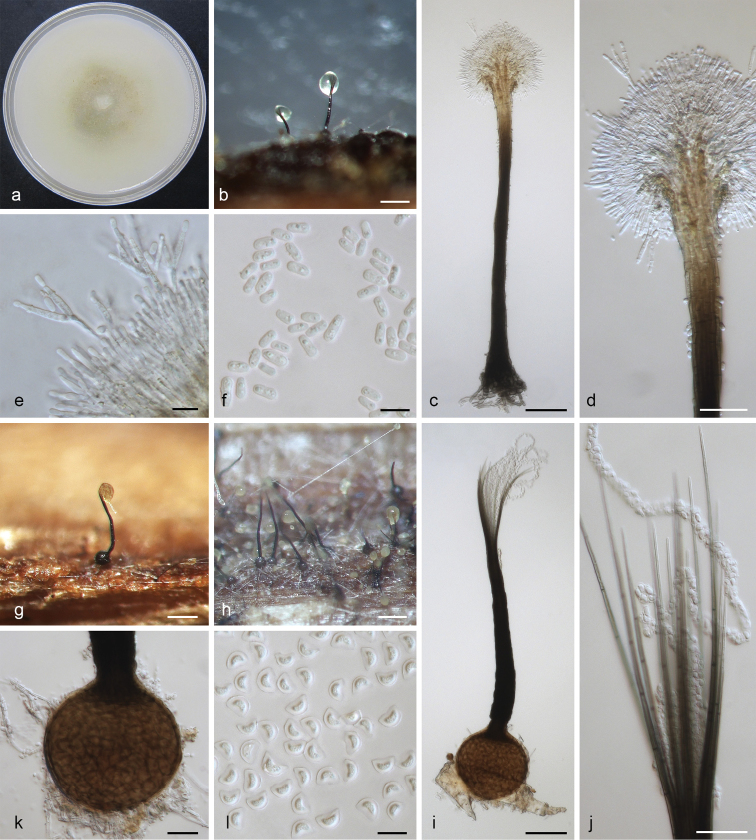
*Leptographium sylvestris* sp. nov. (CMW 34140) **a** fourteen-days old culture on OA with black background **b** synnematous asexual state on wood tissue on WA**c** conidiophore **d** conidiogenous apparatus **e** conidiogenous cells **f** conidia **g–h** the sexual state on wood tissue on WA**i** ascoma **j** ostiolar hyphae **k** ascomatal base **l** ascospores. Scale bars: 100 μm (**b**), 50 μm (**c**), 25 μm (**d**), 10 μm (**e**), 5 μm (**f**), 100 μm (**g**), 100 μm (**h**), 50 μm (**i**), 25 μm (**j**), 20 μm (**k**), 5 μm (**l**).

##### Host trees.

*Pinus sylvestris*, *Picea abies*.

##### Insect vector.

*Ips typographus*.

##### Distributions.

Poland, Finland.

##### Notes.

The Finnish isolate (CMW 23300) was considered by Linnakoski et al. (2012) to be the same undescribed species as the isolates described above as *L. conplurium*. The addition of a newly obtained isolate from Poland in the present study, confirmed that the two isolates represented a distinct taxon, clearly separated from all other species in the complex. This is the only new species for which ascomata were obtained in culture. Single ascospore isolates of this species produced ascomata in culture, suggesting that the species is homothallic. The common characters of sexual states of species in this complex are having ascomata with sheath and ostiolar hyphae on the top of neck. This species differs from others by its fusiform to orange section shaped ascospores and slightly curved neck.

#### Leptographium vescum

Taxon classificationFungiOphiostomatalesOphiostomataceae

(R.W. Davidson) M.L. Yin, Z.W. de Beer & M.J. Wingf. comb. nov.

BAD9BC1A-CBDF-531A-A923-F538C8A4B983

831551

≡ Ceratocystis vesca R.W. Davidson, Mycologia 50: 666. (1958) (Basionym)
≡ Ophiostoma vescum (R.W. Davidson) Hausner, J. Reid & Klassen. Can. J. Bot. 71: 1264. (1993)
≡ Grosmannia vesca (R.W. Davidson) Zipfel, Z.W. de Beer & M.J. Wingf., Zipfel et al., Stud. Mycol. 55: 92. (2006)

##### Type.

USA, Colorado, Fort Collins, from *Ips pilifrons* and *Dendroctonus engelmanni* in *Picea engelmannii*, Jan. 31, 1956, *F.F. Lombard* & *R.W. Davidson*, (**holotype** BPI 595662 = FP 70807, ex-holotype cultures: ATCC 12968 = CBS 800.73 = CMW 34186).

##### Descriptions.

Davidson (1958, p. 666); De Hoog and Scheffer (1984, p. 295, fig. 2); Samuels (1993, p. 16, fig. 1C–F).

##### Host tree.

*Picea engelmannii*.

##### Insect vectors.

*Ips pilifrons*, *Dendroctonus engelmanni*.

##### Distribution.

USA.

##### Notes.

The perithecia of *L. vescum* are smaller than in related species and ascospores are different in shape and size. This species was treated as a synonym of *L. olivaceum* by various authors (Griffin 1968, Olchowecki and Reid 1974, Upadhyay 1981). However, the sequences produced by Hausner et al. (1993, 2000), confirmed by our results, showed that the two species are distinct.

#### Leptographium xiningense

Taxon classificationFungiOphiostomatalesOphiostomataceae

M.L. Yin, Z.W. de Beer and M.J. Wingf. sp. nov.

6A650550-E056-54A7-ADAA-861D04522970

823573

Fig. 8

##### Etymology.

The epithet refers to the locality where the species was first collected.

##### Type.

CHINA, Qinghai Province, from *Picea crassifolia* infested by *Polygraphus poligraphus*, Aug. 2010, *M.L. Yin* & *X.D. Zhou*, (PREM 60916-**holotype**, ex-holotype cultures CBS 136509 = CMW 38891); Qinghai Province, from *Picea crassifolia* infested by *Polygraphus poligraphus*, Aug. 2010, *M.L. Yin*, (PREM 60917-**paratype**, ex-paratype cultures CBS 136510 = CMW 39237).

##### Description.

*Sexual state* not observed. *Conidiophores* macronematous, synnematous, 450–550 μm including conidiogenous apparatus, synnemata occasionally slightly swollen at the base, wider at the stipe, black to brown, expanding branches at the apex, light brown to hyaline, (25–)39–44(–50) μm in width. *Conidiogenous cells* discrete, terminal, percurrent and phialidic proliferation, hyaline, cylindrical, (11–)15–18(–19) × 2–3 μm. *Conidia* hyaline, one-celled, cylindrical to obovoid, (3.9–)4.2–4.5(–4.8) × 1.8–2.4 μm. *Cultural characteristics*: Colonies on OA, spore drops hyaline at first, later becoming light to dark yellowish in the center, hyphae hyaline, appressed and immersed, *synnemata* predominant, aerial mycelium occasionally present on wood tissue, Optimal growth temperature on MEA is 25 °C with radial growth rate 2.0 (± 0.5) mm/d, growth reduced at 10 °C, no growth at 30 °C.

**Figure 8.**
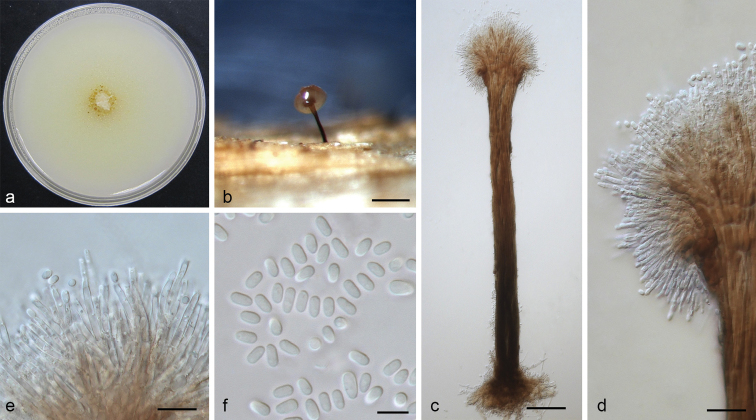
*Leptographium xiningense* sp. nov. (CMW 38891) **a** fourteen-days old culture on OA with black background **b** synnematous asexual state on wood tissue on WA**c** conidiophore **d** conidiogenous apparatus **e** conidiogenous cells **f** conidia. Scale bars: 300 μm (**b**), 50 μm (**c**), 20 μm (**d**), 10 μm (**e**), 5 μm (**f**).

##### Host tree.

*Picea crassifolia*.

##### Insect vector.

*Polygraphus poligraphus*.

##### Distribution.

China.

##### Note.

This species groups closely with *L. conplurium* and *L. erubescens*, but can be distinguished by its dark conidial droplets. In addition, the synnematous conidiophores of this species were shorter, and its conidia were bigger than that of *L. erubescens*.

##### Additional material examined.

CHINA, Qinghai Province, from *Picea crassifolia* infested by *Polygraphus poligraphus*, Aug. 2010, *M.L. Yin* & *X.D. Zhou*, (culture: CMW 39238). Chongqing, from *Pinus armandii* infested by *Dendroctonus armandi*, Nov. 2018, *M.L. Yin*, (culture: SCAU-530). Chongqing, from *Pinus armandii* infested by *Dendroctonus armandi*, Nov. 2018, *M.L. Yin*, (culture: SCAU-531).

## Discussion

Among the five loci used in the phylogenetic analyses, ACT, CAL, and TEF-1α were able to distinguish among all species in the *L. olivaceum* complex. In contrast, TUB sequences could not distinguish between *L. davidsonii* and *L. vescum*. Although ITS2-LSU sequences provided reasonable resolution for species complexes at the genus level, this region could not be used to distinguish among closely related species. Of the five gene regions, TEF-1α had the most variable sites and this is consistent with the results of Yin et al. (2015) for the *L. procerum* complex. This also supports their suggestion that TEF-1α is suitable for use as a barcoding gene for accurate species identification in *Leptographium*.

In this study, we have clarified the previous confusion related to the ex-type isolate of *L. francke-grosmanniae*, and although our phylogenetic data placed it close to the complex, it grouped separated from all other species. This is consistent with its mononematous morphology that distinguishes it from all other species in the complex that produce synnematous asexual states. Furthermore, it is unique in that it does not come from the galleries of a conifer-infesting scolytine bark beetle like the other species, but from the large timberworm beetle, *Hylecoetus dermestoides* (Coleoptera: Lymexylidae), infesting a *Quercus* sp. (Davidson 1971). Some beetles in the latter genus are known to vector ambrosial yeasts (Batra and Francke-Grosmann 1961), but the role and biology of *L. francke-grosmanniae* in these galleries on oak remains unknown. If these beetle ecosystems in hardwoods are explored further, it seems reasonable to expect that additional species related to *L. francke-grosmanniae* could be discovered. These would most likely emerge as a species complex distinct from the *L. olivaceum* complex.

All species in the *L. olivaceum* complex, with the exception of *L. francke-grosmanniae*, share various characteristics. Apart from similar sexual and asexual morphology (as discussed in the introduction), these species are all associated with scolytine bark beetles infesting primarily species of pine (*Pinus*) and spruce (*Picea*). Only *L. davidsonii* has been reported from another conifer genus, namely *Pseudotsuga* (Douglas-fir). However, there is no evidence for strong host or beetle specificity among these fungi. The European spruce bark beetle, *Ips typographus*, for example, infests various species of spruce and pine, and *L. cucullata*, *L. olivacea*, and *L. poloniae*, have been isolated from this beetle or its galleries. Nothing is known regarding the pathogenicity of any of the species in the complex, but Griffin (1968) and Davidson (1958) showed that some species were responsible for the blue-stain of the timber.

In terms of the distribution of species in the *L. olivaceum* complex, our data suggest that most of these taxa are geographically restricted to the continents from which they have been recorded. Four species have been reported only from North America, namely *L. davidsonii*, *L. olivaceapini*, *L. sagmatosporum*, and *L. vescum*, while *L. olivaceum*, *L. erubescens* and four of the new species have been found only in Europe and western Russia. Two of the new species originate from China. Only *L. cucullatum* has been found in Europe and East Asia, specifically Japan.

The results of this study incorporating data for morphology, ecology, and phylogenetic inference based on DNA sequences for five loci have confirmed that the *L. olivaceum* complex is a well-defined species complex in *Leptographium*. Moreover, this integrative approach has been recently employed to resolve lower-level taxonomy in several other groups of fungi such as the Ophiocordycipitaceae (Araújo et al. 2015), Pyronemataceae (Sochorová et al. 2019), Laboulbeniaceae (Haelewaters et al. 2018), Geastraceae (Sousa et al. 2017), and Helvellaceae (Skrede et al. 2017). The combination of multiple properties as independent lines of evidence (e.g., morphology, DNA, substratum, and/or geography) is the way to move forward in fungal taxonomy in general.

## Conclusions

In the present study, DNA sequences for five loci were amplified and used to reconstruct phylogenies for species in the *L. olivaceum* complex. Multilocus phylogenies distinguished clearly among the eight previously described species and also revealed six species: *L. breviuscapum*, *L. conplurium*, *L. pseudoalbum*, *L. rhizoidum*, *L. sylvestris*, and *L. xiningense* that are newly described. TEF-1α was recognized as the best candidate gene to distinguish all species in the complex. For several of the previously known species, problems relating to type specimens were identified, and to resolve these, seven new combinations, two epitypes and three lectotypes have been designated. Following the “one fungus one name” principles, this study provided a model solution to resolving interspecific relationships within the species complexes in the Ophiostomatales. More work should be done on other unresolved species complexes of *Leptographium* and other lineages in the Ophiostomatoid fungi in the future.

## Supplementary Material

XML Treatment for Leptographium breviuscapum

XML Treatment for Leptographium conplurium

XML Treatment for Leptographium cucullatum

XML Treatment for Leptographium davidsonii

XML Treatment for Leptographium erubescens

XML Treatment for Leptographium francke-grosmanniae

XML Treatment for Leptographium olivaceum

XML Treatment for Leptographium olivaceapini

XML Treatment for Leptographium pseudoalbum

XML Treatment for Leptographium rhizoidum

XML Treatment for Leptographium sagmatosporum

XML Treatment for Leptographium sylvestris

XML Treatment for Leptographium vescum

XML Treatment for Leptographium xiningense

## Supplementary materials

Supplementary material 1 The sequence alignment of combined four protein-coding gene regions Data type: phylogenetic data. Explanation note: The alignment was generated from MAFFT V7 Online, and it contained sequences of four protein coding genes (actin, beta-tubulin, calmodulin, and translation elongation factor 1 alpha) of all the isolates in the Leptographium olivacerum complex. https://binary.pensoft.net/file/360789This dataset is made available under the Open Database License (http://opendatacommons.org/licenses/odbl/1.0/). The Open Database License (ODbL) is a license agreement intended to allow users to freely share, modify, and use this Dataset while maintaining this same freedom for others, provided that the original source and author(s) are credited.Mingliang Yin, Michael J. Wingfield, Xudong Zhou, Riikka Linnakoski, Z. Wilhelm de Beer

Supplementary material 2 The sequence alignment of ITS2-LSU gene region Data type: phylogenetic data. Explanation note: The alignment was generated from MAFFT V7 Online, and it contained sequences of Internal transcribed spacer 2 and large-subunit rRNA genes of all isolates used in this study. https://binary.pensoft.net/file/360790This dataset is made available under the Open Database License (http://opendatacommons.org/licenses/odbl/1.0/). The Open Database License (ODbL) is a license agreement intended to allow users to freely share, modify, and use this Dataset while maintaining this same freedom for others, provided that the original source and author(s) are credited.Mingliang Yin, Michael J. Wingfield, Xudong Zhou, Riikka Linnakoski, Z. Wilhelm de Beer
